# A novel mutation in the SRY gene causing 46 XY complete gonadal dysgenesis in a Chinese patient

**DOI:** 10.1186/1687-9856-2013-S1-P189

**Published:** 2013-10-03

**Authors:** Elim Man, Yuet-Ling Tung, Ho-Ming Luk, Fai-Man Ivan Lo, Tak-Sum Stephen Lam, Pik-To Cheung

**Affiliations:** 1 Department of Paediatrics and Adolescent Medicine, Queen Mary Hospital, Li Ka, Shing Faculty of Medicine, The University of Hong Kong; 2 Clinical Genetics Service, Department of Health, Hong Kong, SAR

## Introduction

Complete gonadal dysgenesis with 46 XY karyotype, also known as Swyer-James syndrome, is characterized by complete sex reversal with a female phenotype and unambiguous female external genitalia. Sex-determining region Y (SRY) gene mutations causing loss-of-function of the gene were identified in 10-15% of affected individuals. These individuals also have a high risk of developing tumors such as germinoma and gonadoblastoma in the streaked gonads.

## Case presentation

We report an 18-year old Chinese patient diagnosed with 46 XY complete gonadal dysgenesis. This patient was born with a female phenotype and presented with tall stature, absence of secondary female sexual characteristics and primary amenorrhea in adolescence. The patient is the first child born to healthy and non-consanguineous parents. There was no significant family history. Hormone investigations showed a hypergonoadotrophichypogonadismstate(LH 41.6IU/L, FSH 72.3IU/L, E2<37pmol/L, testosterone 1.6nmol/L). Chromosomal analysis revealed a 46, XY karyotype. Magnetic resonance imaging of the pelvis showed a rudimentary vagina and uterus but no ovarian structure was seen. Laparoscopic exploration revealed bilateral streaked gonads and rudimentary uterus. Both gonads were surgically excised. Gonadal histology revealed a dysgerminoma arising in a residual gonadoblastomain the right gonad while the left gonad showed presence offallopian tube and ovarian stroma but no evidence of malignancy.There was no pelvic lymphadenopathy and evidence of distant metastasis. Disease staging was stage 1a and post-operative chemotherapy was not indicated. Long term hormonal replacement therapy was started.

## Methods and results

Mutation analysis of SRY gene identified a hemizygous c.338C>T sequences variant. This results amissense mutation that change the 113th codon from alanine to valine (Ala113Val). Such amino acid change was located at the high-mobility group (HMG) box of SRY gene that was highly conserved evolutionarily among different species. Together with in-silico study, it was predicted to be pathogenic. This mutation has not be reported in the literature.

## Conclusion

We reported a novel mutation of *SRY* gene that resultedin 46 XY complete gonadal dysgenesis in a Chinese patientandit was complicated with both dysgerminoma and gonadoblastoma.

